# A global dataset of impact forces from submarine landslides on pipelines and cables

**DOI:** 10.1038/s41597-026-06629-1

**Published:** 2026-01-21

**Authors:** Xiaolei Liu, Shuzhou Wei, Xiangshuai Meng, Botao Xie, Xuejian Chen, Xingsen Guo

**Affiliations:** 1 Shandong Provincial Key Laboratory of Marine Engineering Geology and the Environment, Ocean University of China, Qingdao, 266100 China; 2 Sanya Oceanographic Institution, Ocean University of China, Sanya, 572024 China; 3 CNOOC Research Institute Co. Ltd, Beijing, 100028 China; 4 Department of Civil and Urban Engineering, New York University, Abu Dhabi1, 29188 UAE; 5 Department of Civil, Environmental and Geomatic Engineering, University College London, London, WC1E 6BT United Kingdom; 6 Department of Engineering, University of Cambridge, Cambridge, CB2 1PZ United Kingdom

**Keywords:** Natural hazards, Physical oceanography

## Abstract

Submarine pipelines and cables are critical infrastructure components supporting offshore energy production and global communications. These systems are increasingly at risk from submarine landslides, which can generate significant mechanical forces and compromise structural integrity. While various experimental and numerical studies have investigated the interactions between submarine landslides and pipelines or cables, their data are dispersed across disciplines and lack standardization, limiting comparative analysis. Here, we present a curated dataset comprising 864 entries of impact force parameters derived from 24 representative studies. Data were extracted through a systematic literature review covering publications from 1900 to 2025, with an emphasis on works post 2008. Each entry includes key rheological, geometric, and dynamic parameters such as impact velocity, flow type, Reynolds number, and corresponding drag and lift forces. To enhance comparability, we standardized the definitions of peak and stable forces and categorized working conditions based on Reynolds number regimes. This dataset provides a valuable resource for researchers and engineers engaged in risk assessment, offshore infrastructure design, and the modeling of pipeline–landslide interactions.

## Background & Summary

**Fig. 1:**
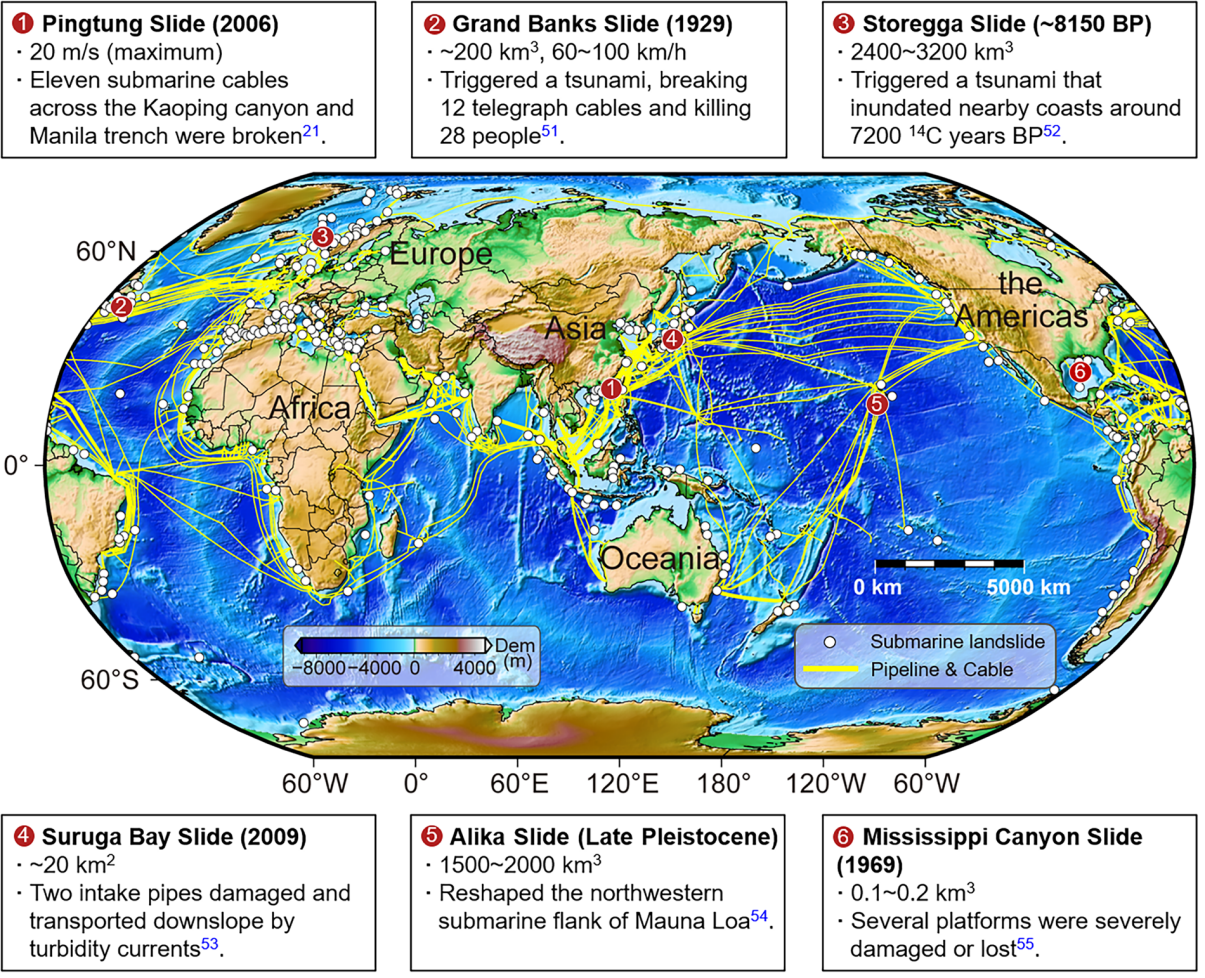
Global distribution of submarine pipeline and cable systems and submarine landslides with representative case studies^21,51–55^.

Submarine pipeline and cable systems refer to submarine oil and gas pipelines and communication cables laid on or beneath the seabed. These systems play a critical role in offshore wind energy development, deep-sea oil and gas exploitation, global data interconnectivity, energy transition, and ecological balance, and thus possess significant economic value and environmental importance^1–4^. Figure 1 illustrates the global distribution of submarine pipeline and cable systems. The total length of submarine oil and gas pipelines worldwide has exceeded 100,000 kilometers^5^, while submarine communication cables extend over 1.4 million kilometers^6^. With the ongoing advancement of intercontinental data interconnection and the increasing number of offshore oil and gas fields as well as deep-water natural gas projects, the scale of submarine pipeline and cable system deployment is expected to continue expanding in the coming years.

In shallow waters, submarine pipelines and cables are typically buried beneath the seabed, whereas in deep waters, they are mostly laid directly on the seabed surface. These systems are often exposed to complex hydrodynamic forces and diverse marine geological conditions, rendering them highly susceptible to damage^7,8^. The primary causes of damage currently include submarine pipeline corrosion, equipment failure, interference from fishing and shipping activities, and submarine geological hazards^9–13^.

Among these hazards, submarine landslides represent a particularly severe threat. They are widely distributed across nearshore deltas, continental shelves, continental slopes, and deep-sea basins^14–16^, as shown in Fig. 1. Since submarine pipeline and cable systems inevitably traverse landslide-prone areas, they are especially vulnerable to damage from such events^17–19^. For instance, in March 1977, a submarine oil pipeline operated by Texaco in the United States was damaged by seabed mud sliding, resulting in a spill of 2,100 gallons of crude oil^20^. In 2006, the Pingtung earthquake in Taiwan triggered turbidity currents in a submarine canyon, severing multiple submarine communication cables and causing significant economic losses. Between 2009 and 2010, the same region experienced several submarine landslides due to flooding and seismic activity, leading to repeated damage to submarine cables^21,22^. Therefore, accurate analysis and prediction of the impacts of submarine landslides on submarine pipeline and cable systems is essential for optimizing engineering design and ensuring the safe operation of offshore infrastructure.

Studies have shown that the impact force exerted by submarine landslides on submarine pipeline and cable systems can be decomposed into three components: the drag force *F*_D_, acting parallel to the landslide direction; the lift force *F*_L_, acting in the vertical direction; and the axial force *F*_A_, aligned with the axis of the cable^9^. During the interaction between landslides and cables, the flow field structure undergoes substantial changes, leading to significant variations in both the velocity field and shear rate, and resulting in a nonlinear evolution of the impact forces^23^. This complex process is influenced by a range of factors. For instance, when the landslide first makes contact with the cable, a large pressure gradient can cause the drag force to spike sharply, reaching a peak value^24^. Under high Reynolds number conditions, vortex shedding from the trailing edge of the flow may induce strong fluctuations in the lift force during the quasi-steady stage^25^.

At present, research on the impact of submarine landslides on pipelines and cables primarily relies on physical model experiments and numerical simulation methods. This topic spans multiple disciplines, including geotechnical mechanics, fluid mechanics, marine geology, and structural mechanics. Existing studies have contributed to the continuous development and refinement of various theoretical frameworks–such as geotechnical mechanics, fluid mechanics, and hybrid geotechnical–fluid mechanics^26–28^. Significant progress has also been made in experimental techniques, including large-scale flume tests and geotechnical centrifuge modeling^9,29–32^. In addition, numerical approaches such as computational fluid dynamics (CFD), the material point method (MPM), and smoothed particle hydrodynamics (SPH) have been widely adopted to simulate the complex interaction between landslides and pipelines^26,33–36^.

However, due to the diversity of research objectives, inconsistencies in methodological frameworks, and disciplinary differences among researchers, comparative analysis and integrated application of existing findings remain challenging. To address this gap, the present study establishes a dataset of submarine landslide-induced impact forces on pipelines and cables based on a comprehensive review of existing literature, systematically compiling 864 sets of quantitative data on the impact forces exerted by submarine landslides on cables. This dataset is intended to provide a more structured and comprehensive foundation for future research, engineering design, and risk assessment.

## Methods

This study developed a dataset of submarine landslide impact forces on pipelines and cables through systematic literature review and data compilation. The overall workflow consists of the following key stages: literature retrieval, initial screening and evaluation, data extraction, and aggregation. The specific procedures involved in each stage are detailed below.

### Data sources

In this study, the full Web of Science (WoS) database was used to retrieve relevant literature on the interaction between submarine landslides and pipelines and cables. The search terms included combinations of “submarine landslide”, “submarine slide”, “submarine slump”, “submarine debris flow”, “turbidity flow” “gravity current” or “debris flow” with “pipeline” “cylinder” or “cable”, and the document type was limited to articles. All records containing any of the above terms in the title, keywords, or abstract were included in the initial search scope. The search covered the period from 1900 to 2025, with particular emphasis on literature published since 2008. The last update of the literature data was completed on November 30, 2025.

Following an initial screening, we identified a total of 868 valid records and subsequently selected over 100 publications that were highly relevant to the topic of submarine landslide impact forces on pipelines and cables for further evaluation. Based on a comparative assessment of research methods, parameter completeness, and data accessibility, 24 representative studies were ultimately retained for constructing the dataset^7,9,19,23,25,26,30,31,34–49^. All source publications are explicitly cited in the dataset at the level of individual data entries (see the “source_ref” column in the “main_dataset” sheet). These studies encompass the major research approaches, including physical model experiments and numerical simulations, and cover typical types of submarine landslides such as debris flows, turbidity currents, and gravity currents. The associated parameter space spans a wide range of Reynolds numbers and operational conditions, providing the dataset with broad coverage and strong representativeness.The primary criteria for literature selection were as follows: (1) the study employed numerical simulation or physical modeling and reported quantifiable data on pipeline loads or structural responses; (2) key control factors governing impact forces in the pipeline–environment interaction were systematically examined; (3) the work was grounded in established theoretical frameworks of geotechnical mechanics and/or fluid dynamics.

### Data extraction

After identifying the target literature, this study systematically classified and extracted data related to submarine landslide impact forces. The original data were predominantly available in the form of tabulated values and graphical plots. A total of 864 valid data entries were compiled and subsequently normalized based on a unified parameter definition system, as detailed in Table 1. This system covers the principal factors influencing landslide-induced impact forces, including the structural characteristics of pipelines and cables, the rheological properties of the landslide material, and the spatial configuration between the landslide body and the Submarine pipeline and cable systems during their interaction.

**Table 1:** List of parameters used in the dataset.

Parameter	Unit	Description
Sample ID	—	An integer identifier used to uniquely label each sample.
Reference ID	—	An integer identifier that points to relevant reference.
Flow Type	—	A categorical parameter that denotes the flow type of submarine landslides.
*U*_∞_	m/s	Impact velocity of the submarine landslide
*ρ*	kg/m^3^	Density of the submarine landslide
*ρ*_0_	kg/m^3^	Density of the ambient surrounding fluid, if not explicitly specified in the original study, ρ₀ is assumed to be 1000 kg/m³.
(*ρ-ρ*_0_)/*ρ*_0_	—	Density difference, defined as the relative density contrast between the submarine landslide and the ambient fluid, expressed as(ρ-ρ0)/ρ.
*D*	mm	Diameter of the pipeline
*τ*_y_	Pa	Yield stress of the submarine landslide
*K*	Pa·s^n^	Consistency coefficient
*n*	—	Fluidity index
*τ*	Pa	Shear stress of the submarine landslide
$\dot{\gamma }$	s^−1^	Shear rate of the submarine landslide
*T*	°C	Temperature of the submarine landslide
*s*_u_	kPa	Shear strength of the submarine landslide
*Re*_non - Newtonian_	—	Reynolds number of non-Newtonian fluids
*H*	mm	Span height of the pipeline, defined as the vertical distance from the bottom of the pipeline to the seabed.
*H*_C_	mm	Landslide cover thickness above the submarine pipeline (Note: The distance between the landslide’s top surface and the pipeline’s upper surface)
*H*/D	—	Span height ratio, defined as the dimensionless ratio between the span height of the pipeline and its diameter.
*k*_s1_	mm	Roughness of the pipeline surface (Note: This study adopts an equivalent sand grain model, assuming that the surface roughness of the pipeline is uniformly distributed.)
*k*_s2_	mm	Roughness of the seabed
*θ*	°	Angle of impact, defined as the angle of the pipeline’s axis in the counterclockwise direction.
*Ut/D*	—	Dimensionless impact distances
*ψ*_c_	—	Ratio of the submarine landslide cover thickness to the diameter of the submarine pipeline, expressed as *H*_C_/D.
*F*_D_	N	Drag force, defined as the force acting along the run-out direction of the submarine landslide and normal to the pipeline axis.
*F*_D-P_	N	Peak drag force
*F*_D-S_	N	Stable drag force
*C*_D_	—	Drag coefficient, a dimensionless coefficient.
*C*_D-P_	—	Peak drag coefficient
*C*_D-S_	—	Stable drag coefficient
*F*_L_	N	Lift force, defined as the force acting perpendicular to both the pipeline axis and the run-out direction of the submarine landslide.
*F*_L-P_	N	Peak lift force
*F*_L-S_	N	Stable lift force
*C*_L_	—	Lift coefficient, a dimensionless coefficient.
*C*_L-P_	—	Peak lift coefficient
*C*_L-S_	—	Stable lift coefficient

Peak and steady-state loads exerted by submarine landslides on submarine pipeline and cable systems are key parameters for engineering design. However, inconsistencies in the definitions of “peak” and “steady-state value” across different studies hinder standardized quantitative comparisons. In this study, all literature data containing force-time curves were processed through a unified quantitative standardization procedure, ensuring consistent extraction of peak and steady-state impact forces. For studies that report impact forces only in tabulated form, the original values were retained.

Specifically, the peak impact force is defined as the global maximum of the force-time curve. The steady-state value is identified under the following conditions: when the curve enters a stable regime or exhibits periodic oscillations, the steady-state value is calculated as the mean of the maximum and minimum forces within that regime. If the curve does not exhibit a stable or periodically oscillatory behavior—i.e., its relative fluctuation range (defined as the percentage ratio of the difference between the maximum and minimum values to their mean) exceeds 10%—the steady-state value is marked as “NaN” and excluded from normalization. This approach prevents subjective or physically unjustified interpretations.

**Fig. 2:**
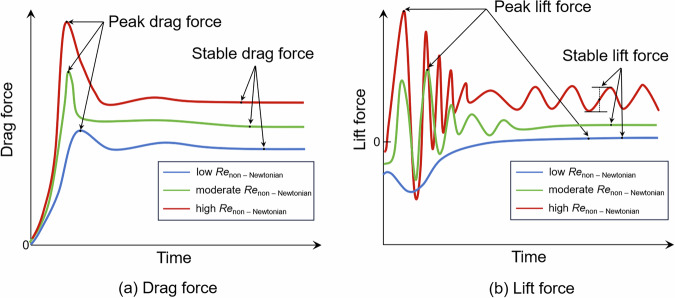
Definition of the peak value and stable value of impact forces^46^: (**a**) Drag force; (**b**) Lift force.

Furthermore, the operating conditions are classified into three categories according to the Reynolds number of the non-Newtonian flow: low Reynolds number (*Re*_non-Newtonian_ < 10), medium Reynolds number (10 ≤ *Re*_non-Newtonian_ < 50), and high Reynolds number (*Re*_non-Newtonian_ ≥ 50)^46^. The force-time responses of pipelines exhibit distinct characteristics across these regimes. By applying unified definitions for peak and steady-state impact forces, this study establishes a standardized representation of pipeline mechanical responses. As an illustration, Fig. 2 presents typical trends in drag and lift forces under different flow conditions.

**Fig. 3:**
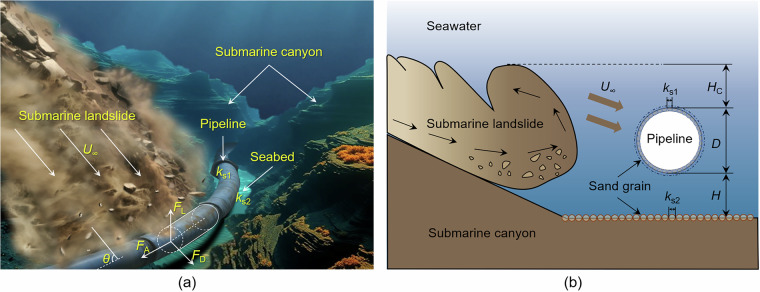
Schematic illustration of submarine landslide impact on pipeline and cable systems: (**a**) Three-dimensional conceptual diagram; (**b**) Two-dimensional cross-sectional view highlighting key parameters (*U*_∞_, *D*, *H*, *H*_C_, *k*_s1_ and *k*_s2_) involved in the landslide–pipeline interaction.

Figure 3 provides a generalized illustration of the impact of submarine landslides on pipelines and cables, incorporating multiple parameters to comprehensively reflect their effects. Figure 3(a) presents a three-dimensional schematic of a submarine landslide impacting a suspended pipeline system within a submarine canyon, offering a clear understanding of the key variables governing the interaction between the landslide and the pipeline. Figure 3(b) depicts a two-dimensional cross-sectional view, annotating the primary geometric and flow parameters included in the dataset, such as span height, roughness, and landslide cover thickness. Among these parameters, the definition of span height varies across the literature: while some studies define it as the vertical distance from the pipeline center to the seabed^35,39^, this study standardizes it as the vertical distance from the bottom of the pipeline to the seabed.

## Data Records

We present the dataset in Figshare (10.6084/m9.figshare.29877113)^50^. It is provided in Excel format and comprises 864 standardized records extracted from 24 representative studies. The dataset consists of four worksheets: parameters, which lists all variables used in the dataset along with their definitions and units; flow_type_glossary, which summarizes the terminology and classification of submarine landslide flow types reported across different studies; main_dataset, which serves as the primary data table and contains all entries with standardized parameters and their corresponding reference identifiers; and references, which provides complete citation information for the source literature associated with each data entry.

## Technical Validation

### Error control

Several studies report force-time data only in graphical form, which prevents direct use of the original numerical values. To extract these data, this study employed the Digitizer tool in OriginLab, which digitizes curves based on coordinate mapping. Potential sources of error in this process include: (1) limited image resolution, which may obscure axis scales or individual data points, and (2) overlapping data points in densely plotted regions, making accurate identification more challenging.

To reduce uncertainties associated with resolution and point overlap, the original images were magnified as much as possible during digitization, and data points were consistently selected at the center of the plotted markers to ensure precise positioning. No interpolation, smoothing, curve fitting, or any procedure that could alter the original data structure was applied, nor were curves reconstructed or modified. All extracted values were retained to four decimal places to maintain consistency in data formatting.

These measures ensured that the integrity of the original force-time data was preserved while keeping manual digitization errors within a negligible range. Although manual extraction remains a potential source of uncertainty, its influence has been minimized and is fully documented through the transparent and traceable design of the dataset.

### Data traceability and transparency

The data used in this study are exclusively from published, peer-reviewed literature. These studies encompass laboratory flume tests, centrifuge model tests, and numerical simulations (CFD, LES, etc.). Each record in the dataset includes the publication number and parameter definitions, allowing users to directly trace back to the original study.

Through parameter completion and normalization, rigorous manual extraction error control, and data support from literature, the dataset constructed for this study not only integrates disparate data sources but also ensures the integrity and scientific validity of the results.

## Data Availability

The dataset generated and presented in this study is publicly available on Figshare under the 10.6084/m9.figshare.29877113. All data files, including the main dataset and supplementary sheets, can be accessed without restriction.

## Code availability

No custom code was developed or utilized in this study. All data were manually extracted from the literature and processed using standard spreadsheet software, such as Microsoft Excel.
